# Rapid relaxation of pandemic restrictions after vaccine rollout favors growth of SARS-CoV-2 variants: A model-based analysis

**DOI:** 10.1371/journal.pone.0258997

**Published:** 2021-11-24

**Authors:** Debra Van Egeren, Madison Stoddard, Alexander Novokhodko, Michael S. Rogers, Diane Joseph-McCarthy, Bruce Zetter, Arijit Chakravarty

**Affiliations:** 1 Department of Systems Biology, Harvard Medical School, Boston, MA, United States of America; 2 Department of Data Science, Dana-Farber Cancer Institute, Boston, MA, United States of America; 3 Stem Cell Program, Boston Children’s Hospital, Boston, MA, United States of America; 4 Fractal Therapeutics, Cambridge, MA, United States of America; 5 Department of Mechanical Engineering, University of Washington, Seattle, WA, United States of America; 6 Vascular Biology Program, Boston Children’s Hospital, Boston, MA, United States of America; 7 Department of Biomedical Engineering, Boston University, Boston, MA, United States of America; Texas A&M University College Station, UNITED STATES

## Abstract

The development and deployment of several SARS-CoV-2 vaccines in a little over a year is an unprecedented achievement of modern medicine. The high levels of efficacy against transmission for some of these vaccines makes it feasible to use them to suppress SARS-CoV-2 altogether in regions with high vaccine acceptance. However, viral variants with reduced susceptibility to vaccinal and natural immunity threaten the utility of vaccines, particularly in scenarios where a return to pre-pandemic conditions occurs before the suppression of SARS-CoV-2 transmission. In this work we model the situation in the United States in May-June 2021, to demonstrate how pre-existing variants of SARS-CoV-2 may cause a rebound wave of COVID-19 in a matter of months under a certain set of conditions. A high burden of morbidity (and likely mortality) remains possible, even if the vaccines are partially effective against new variants and widely accepted. Our modeling suggests that variants that are already present within the population may be capable of quickly defeating the vaccines as a public health intervention, a serious potential limitation for strategies that emphasize rapid reopening before achieving control of SARS-CoV-2.

## Introduction

The ongoing COVID-19 pandemic has taken a heavy toll on global health, prosperity, and stability. Within the United States, the recent deployment of several highly efficacious vaccines has led to a wave of optimism, with widespread coverage in the lay press [1–7] heralding a return to normalcy in the coming months.

The emergence of immune-evading variants of SARS-CoV-2 poses a potential risk to a return to normalcy. A number of newly emerged variants have been demonstrated experimentally to be more capable of infecting cells, spreading between hosts, and/or evading natural immunity, vaccines and therapeutics, compared to the original wild-type (WT) SARS-CoV-2 [8–10]. Once variants emerge within a population, they tend to expand rapidly and deterministically due to natural selection and dominate the local viral population in a relatively short period of time [11]. These variants have in a number of cases been associated with more severe disease outbreaks, and some variants have been demonstrated to be less susceptible to certain vaccines [8, 9, 12, 13].

As public health authorities struggle with the problem of vaccine hesitancy, a strategy of trading away non-pharmaceutical interventions (NPIs, e.g., mask wearing, reduced mobility and social distancing) to incentivize higher vaccine uptake has emerged, both in the United States and elsewhere. Encouraged by data showing that vaccination protects against symptomatic and asymptomatic infection and against hospitalization due to COVID-19, the CDC recently stated that fully vaccinated individuals can resume normal activities without masks or physical distancing [14]. This change in CDC guidance has been mirrored at state and local levels, as many states have lifted their mask mandates [15], and social distancing and mask-wearing within the United States have dropped rapidly in recent weeks [16].

Implicit in the strategy of relaxing restrictions to encourage vaccine uptake is the expectation that sufficiently high levels of uptake would lead to the development of herd immunity. Such herd immunity, where transmission of the virus is blocked by high levels of immunity within the population, has been shown to be within reach given the high efficacy rates of some of the vaccines [17]. A second scenario supporting the use of the above strategy is when high levels of vaccine acceptance coupled with gradual reopening lead to the stochastic extinction of potentially vaccine-evading variants of the virus due to drift, thus driving transmission down and allowing relaxation of further interventions. A case can be made that this public health outcome may in fact have been achieved successfully in some parts of the world, for example in Israel and Gibraltar [18–20], where high rates of vaccination were followed by a gradual relaxation of restrictions. Notably, the extermination of problematic viral variants due to stochastic events may be feasible even in the absence of herd immunity, provided viral transmission is low enough [11, 21].

Unfortunately, in a situation where vaccine-evading variants are circulating widely within the population as the vaccine is deployed, evolution and infectious disease dynamics would be expected to play out quite differently. In the presence of a widely deployed vaccine, natural selection will act to enrich for the vaccine-resistant variants. Under conditions of high transmission, with a large pre-existing vaccine-resistant viral population, this can lead to a scenario where variants substantially reduce vaccine efficacy on a population level.

In this work, we examine the practical implications of the strategy of vaccinating widely and attempting a return to pre-pandemic conditions, using as our example the situation in the United States in the coming months. As of early June 2021, the United States has vaccinated 46% of its adult population [22] and vaccines are expected to reach 70% of the adult population by July [23]. As 25% of the United States population consists of children, and vaccine acceptance among the 12–15 year old population (newly eligible for the vaccine) is expected to be lower than acceptance for adults [24], one can roughly estimate that the fraction of the total population vaccinated by July would be around 50%. There are five variants of concern as defined by the CDC present at appreciable frequencies in the United States at present (see S1 Table for details): B.1.427 and B.1.429 with a transmissibility 20% greater than ancestral Wuhan strain (Wuhan-Hu-1, referred to here as “WT”); Alpha (B.1.1.7, the “United Kingdom” variant), with a transmissibility approximately 60% higher than wild type [9] and vaccine efficacy reduction of approximately 10% against the Pfizer vaccine [25]; Beta (B.1.351, the “South African” variant), with a transmissibility approximately 50% higher than WT [26] and vaccine efficacy reduction of approximately 25% against the Pfizer vaccine [25]; and Gamma (P.1, the “Brazilian” variant), with a transmissibility approximately 100% higher than WT [27] and a 32% reduction of immunity induced by WT infection [27]. As of April 10th, 2021, Alpha, Beta, and Gamma constitute 60%, 1% and 3.7% of all US infections [28] (S1 Table). There are other variants that have emerged recently, such as Delta (B.1.617, the “Indian” variant) that are not yet fully characterized and may emerge as a threat during the timeline examined here, but we have not considered these.

We have used a model incorporating the dynamics of immune-evading variants to ask the question: “Do the expected levels of vaccine coverage in the US allow us to return to normal without suppressing viral transmission first?” Using an extended Susceptible-Infected-Recovered (S-I-R) epidemiological model with two or more competing variants with simulation conditions mirroring the current situation in the United States, we modeled the impact of vaccine-evading variants on the course of the COVID-19 pandemic in the presence of vaccines.

## Results

**Fig 1:**
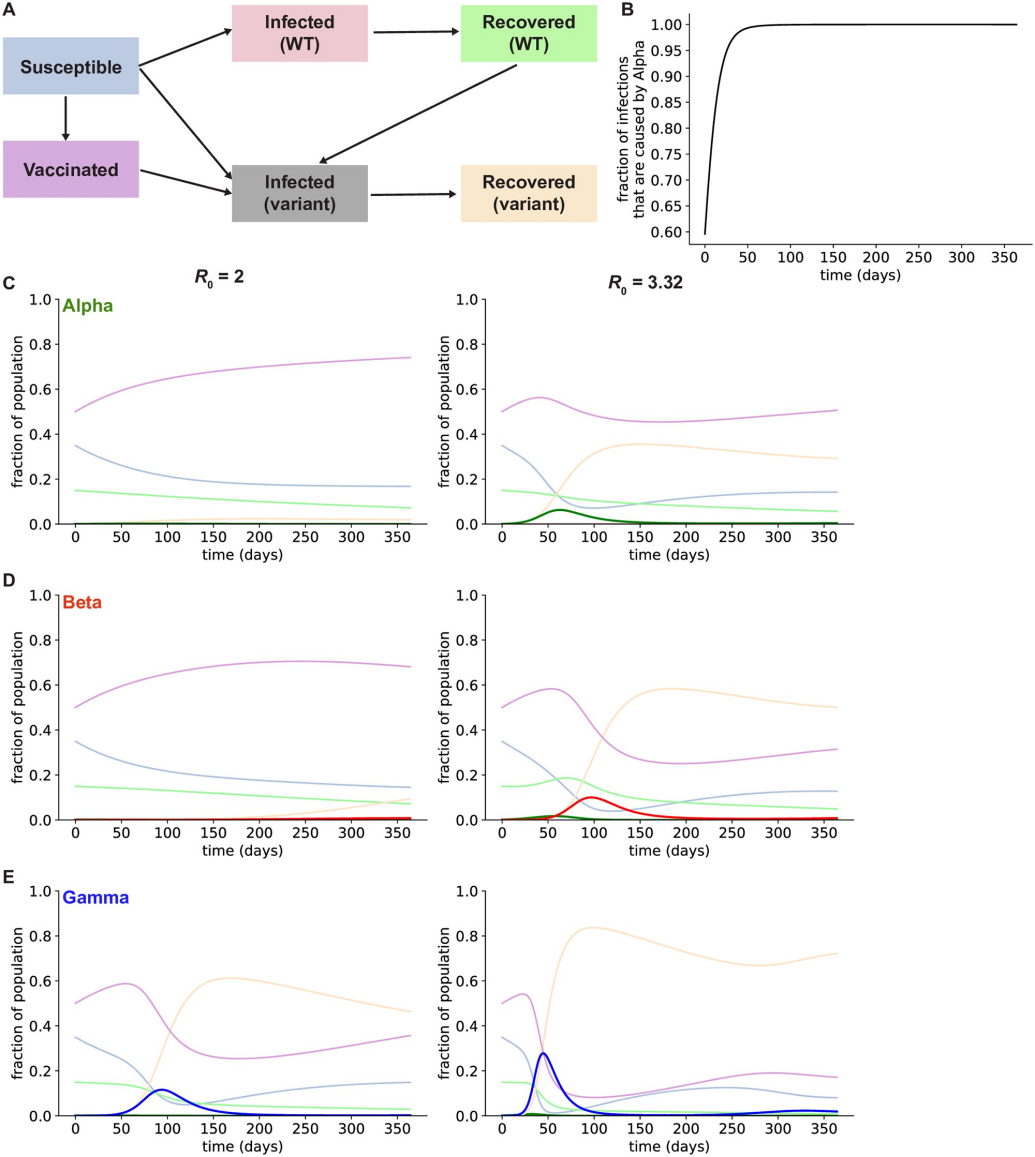
Existing SARS-CoV-2 variants are likely to cause a surge of infections in the US after reopening. A: Structure of the extended S-I-R model. Susceptible individuals can be infected with WT or variant virus, while individuals with immunity to the WT virus can only be infected by variants. Individuals can also lose protective immunity and rejoin the susceptible population (not shown in this schematic). B: Fraction of active infections caused by the Alpha variant, assuming an *R*_0_ of 3.32 and that no additional variants are present in the population. C: Simulated frequencies of infections caused by the Alpha variant (green) for two different values of *R*_0_, assuming it is the only variant present in the population. D: Simulated frequencies of infections caused by the Gamma variant (blue), assuming all other infections are caused by the Alpha variant (green). E: Simulated frequencies of infections caused by the Beta variant (red), assuming all other infections are caused by the Alpha variant (green). For C-E, frequencies of other compartments are plotted in the colors used in the schematic in A.

To better understand the impact of pre-existing variants on a vaccinated population, we conducted epidemiological modeling in a hypothetical setting with only three variants (Alpha, Beta, and Gamma) present. We used an extended S-I-R model with additional infected and recovered compartments for one or more variants with different transmissibilities and conferring different degrees of immunity to other variants (Fig 1A). This model also includes a separate population of vaccinated individuals and accounts for waning immunity over time for both vaccinated and previously infected individuals (see Methods for details). Specifically, we assumed that vaccinated individuals could not be infected by WT virus but could be infected by variants. However, vaccination and previous infections with other variants confers partial protection against variant infection. Additionally, we assumed that vaccinated individuals who are infected with variants can transmit the infection at the same rate as individuals who were infected but not previously vaccinated. Although vaccinated individuals who are infected with WT SARS-CoV-2 have approximately 10-fold lower viral loads than unvaccinated individuals [29], the size of the viral inoculum required to start an infection is many orders of magnitude lower than the standing viral population in an infected individual [30]. Similarly, a recent study demonstrated that a reduction in viral load of 4 orders of magnitude corresponds to only a 50% decrease in transmissibility [31, 32], suggesting that vaccinated individuals with breakthrough infections can still transmit SARS-CoV-2. Parameter values used in the simulations are given in the supplement (S1 and S2 Tables).

Initially, we assumed that a single variant (Alpha, Beta, or Gamma) was present in the population at the frequency estimated in the US population on April 10th, 2021. We simulated the spread of three existing SARS-CoV-2 variants (Alpha, Beta, and Gamma) in the US if the contact rate (the maximum rate at which infected individuals can spread the infection, as dictated by their interactions with others) is allowed to return to pre-pandemic levels (*R*_0_ = 3.32). Assuming a moderate level of vaccine coverage similar to current conditions in the US (50% of the population is initially fully vaccinated), we found that the Alpha variant will quickly expand and become responsible for nearly all infections (Fig 1B and 1C), as it did in the UK [9]. However, the other two variants, which are more capable of infecting individuals who were vaccinated or have been previously infected with WT virus, will cause a spike of infections months after the return to normal contact levels. If only Gamma and Alpha are present in the population, Gamma will cause a wave of infections affecting >80% of the population, including those who have been vaccinated (Fig 1D). Without Gamma, the Beta variant will still cause a wave of infections, although it will occur later and affect fewer individuals (Fig 1E). If contact rates remain low (*R*_0_ = 2), the Alpha and Beta variants are not predicted to cause a clear spike in the number of infections (Fig 1C and 1D). However, if Gamma is present, it will cause a rise in COVID-19 cases due to its increased transmissibility and immune evasion properties, even at lower contact rates (Fig 1E).

**Fig 2:**
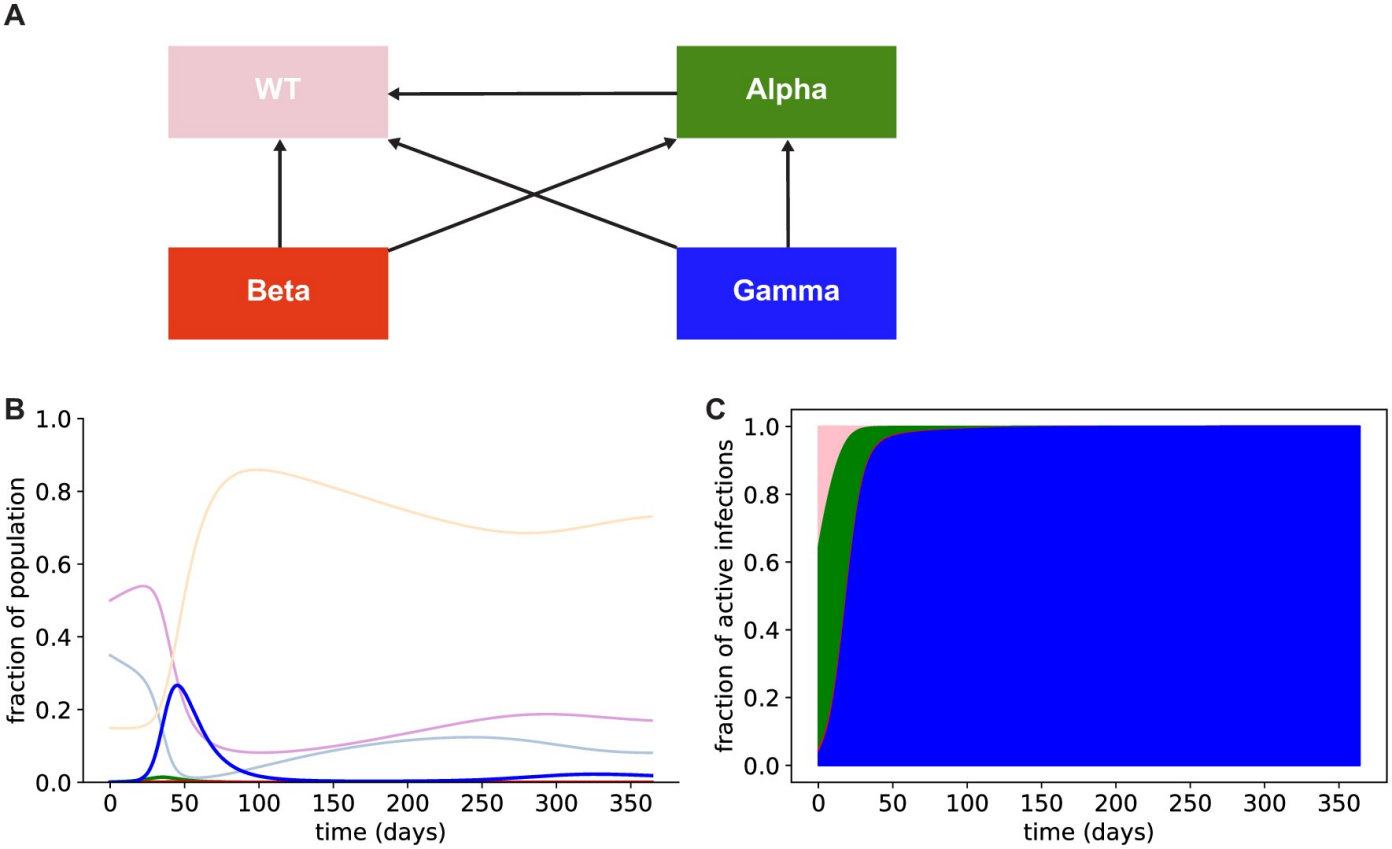
The Gamma variant is likely to outcompete other existing variants and drive a surge of infections even in a population with high WT immunity. A: Schematic showing cross immunity between variants used in our simulations. Arrows show which variants can infect individuals with immunity to other variants. For example, the arrow from Beta to WT indicates that Beta can infect individuals with immunity to the WT virus acquired by prior infection or vaccination. B: Fraction of individuals infected with SARS-CoV-2 variants (colors as in A), simulated over 1 year. Susceptible (light blue), vaccinated (light purple), and recovered from SARS-CoV-2 infection with any viral genotype (orange) fractions are also plotted. C: Fraction of active infections caused by each existing SARS-CoV-2 variant over the first year (colors as in the schematic in A).

We then further extended the S-I-R model shown in Fig 1A to include three competing variants simultaneously, and we used this extended model to simulate the case in which all three variants were present to investigate possible clonal interference between the variants. Assuming all three variants are initially present in the population (at the frequencies estimated by the CDC as of 4/10/21), if a full return to pre-pandemic conditions is attempted after vaccination, Gamma will become the dominant strain in the US within a matter of months (Fig 2). This surge in variant infections is predicted to lead to hundreds of millions of COVID-19 cases, as well as millions of COVID-19 fatalities (Table 1). Predicted fatalities shown in Table 1 were calculated using a base infection fatality rate of 0.68% [33] (Methods), and an additional 46% reduction in risk of death given infection for vaccinated individuals (Methods), as reported for the Pfizer vaccine and wild-type SARS-CoV-2 [34]. Variant infections (notably, Gamma) will be responsible for the vast majority of cases and will increase the total infection burden approximately 100-fold (Table 1). These results suggest that, in the presence of vaccine-evading strains, a return to pre-pandemic levels of contact following vaccination is likely to result in profound mortality and morbidity.

**Table 1:** A return to pre-pandemic levels of mobility following vaccination is made infeasible by the presence of vaccine-evading variants.

Scenario	Total infections (millions)	Total fatalities (millions)
Only WT	3.5	0.02
WT + Alpha	148.3	0.85
Alpha + Beta	269.7	1.4
Alpha + Gamma	370.1	1.8
WT + all 3 variants	373.7	1.9

Predicted SARS-CoV-2 infection and mortality burdens over one year upon a return to pre-pandemic levels conditions (*R*_0_ = 3.32), in the United States. The predicted fatalities were calculated using a base infection fatality rate of 0.68% and assuming a 70% reduction in risk of death given infection for vaccinated individuals.

**Fig 3:**
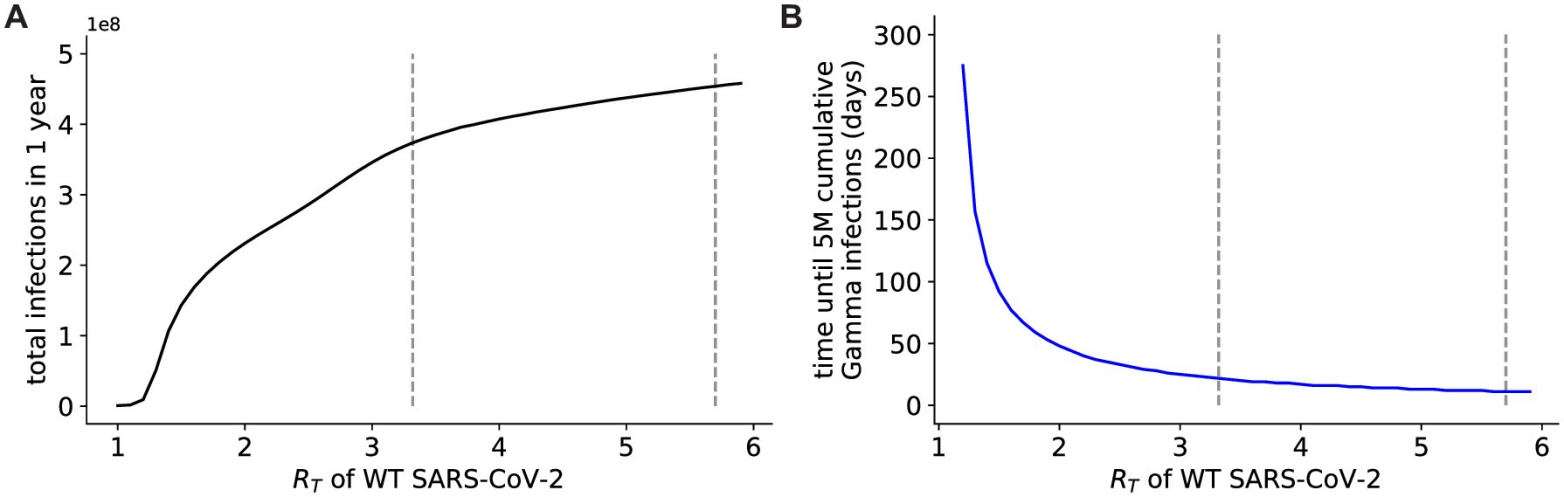
Reopening with an increased infection contact rate will lead to high total infection burden and rapid transmission of the Gamma variant in the US. A: Total infection burden for all variants over different contact rates corresponding to different *R*_*T*_ values for WT SARS-CoV-2. B: Length of time from the start of the simulation until 5 million individuals have been infected with the Gamma variant, given different contact rates corresponding to different *R*_*T*_ values for WT SARS-CoV-2. Vertical dashed lines in both panels correspond to *R*_0_ values measured at the beginning of the pandemic (3.32 and 5.7), before infection control policies were implemented.

Continuing to suppress the overall SARS-CoV-2 reproduction number (*R*_*T*_) with NPIs can delay the surge of variant infections and reduce overall infection burden (Fig 3). The basic reproductive number *R*_0_ in Wuhan under normal contact levels for the WT strain was estimated to be 3.3236 [35], though estimates of this value have been as high as 5.737 [36]. Reducing this rate to approximately 1 for WT SARS-CoV-2 substantially suppresses the spread of all three variants, limiting the number of total infections over the next year (Fig 3).

**Fig 4:**
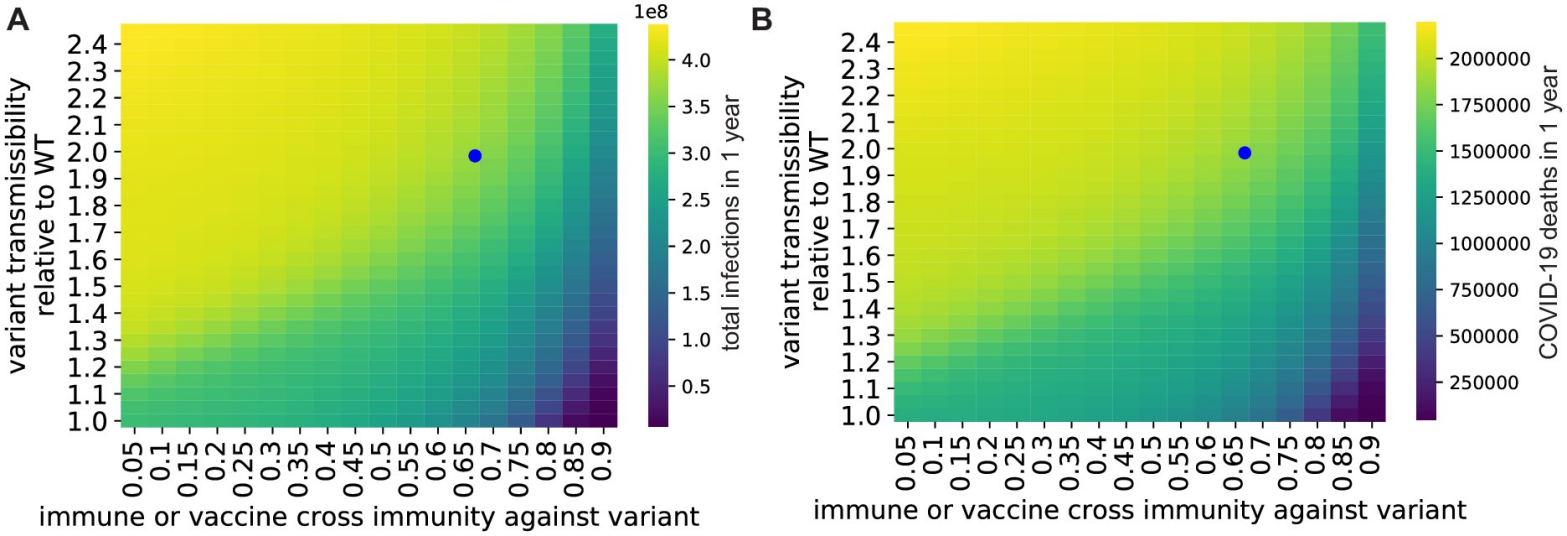
Variants that have increased transmissibility and/or immune evasion ability can cause substantial disease spikes in vaccinated communities after a return to pre-pandemic contact levels. A. Total new SARS-CoV-2 infections within one year after introduction of variants with different values for transmissibility and cross immunity with WT. B. Predicted COVID-19 fatalities within one year. For both panels, the variant was initially responsible for 3.7% of infections (the initial frequency of the Gamma variant used elsewhere in this study) and all other parameter values are those given in S2 Table. The blue dot in both panels represents the Gamma variant parameters.

We also performed parameter value sweeps to determine how our conclusions depended on two key variant parameters: the transmissibility of the variant relative to WT and the cross immunity of the variant with WT SARS-CoV-2 (Fig 4). Using the initial frequency for Gamma in the population as the starting variant frequency, and an *R*_0_ value of 3.32, we found that variants with modest transmission advantages (>50% increase) and/or immune evasion potential (<80% vaccine efficacy against the variant) will lead to high infection burdens (>300 million new infections) in the US over the course of one year. These sweeps suggest that our conclusions are robust to errors in the published estimates for transmissibility and cross immunity. Furthermore, these sweeps suggest that future variants that are more transmissible and/or immune evading than Gamma have the potential to cause larger outbreaks under high contact rate conditions in vaccinated populations.

Taken together, our findings indicate that a relaxation of restrictions will favor the rapid spread of pre-existing partially immune-evading strains (such as Gamma in our example here), in a manner that is strongly dependent on the contact rate.

## Discussion

In this work we demonstrate that a return to pre-pandemic conditions following modestly high levels of vaccination will efficiently select for pre-existing vaccine-evading viral variants within the population, causing a high level of infection and potentially death.

While these results paint an alarming picture, they are not intended as a specific prediction of the future. Rather, we sought to illustrate a scenario in which the pandemic may- counterintuitively- rebound after reopening despite widespread vaccination. In particular, we focused on the consequences of a return to pre-pandemic conditions after moderate to high levels of vaccination, while vaccine-evading variants are still circulating in the population. Our findings suggest that, under certain conditions, this strategy will lead to strongly negative public-health consequences in the short term.

While this manuscript was under review, a number of other publications were released, supporting our findings. The idea that variants will significantly impact the timing of the end of the pandemic- even after vaccination- has become increasingly obvious, driven by current events (Fontanet et al [37] provides an excellent set of policy recommendations in this regard). Modeling studies focused on the UK [38], Italy [39], and Portugal [40] have also pointed out the flaw in the strategy of relaxing restrictions during the vaccination campaign, as relevant to each of those countries. These studies, which used the assumptions of durable natural and vaccinal immunity, examined the impact of relaxing restrictions on the course of the pandemic driven by the ancestral strain. While our results are qualitatively very similar to these findings, the higher transmissibility and immune evasion potential of the variants, coupled with waning immunity, leads to significantly higher estimates for morbidity and mortality in our work.

Our work has several important limitations. First, the simulations of the United States shown here are examples based on the currently available estimates for viral parameters such as transmissibility and vaccine evasion. Those estimates are preliminary and may change as more information becomes available. The results here depend heavily on the estimates for transmissibility for the immune-evading strains (Gamma and Beta), and these estimates are vulnerable to experimental error [41]. Thus, while our work suggests, for example, that Gamma is likely to pose a public health threat in the coming months within the United States if contact rates return to pre-pandemic levels, uncertainty around viral parameters for Gamma prevents us from making a definitive statement. Second, a number of key parameter values in our system are currently unknown: for example, vaccine impact on the transmission of viral variants, the degree of cross-protection against reinfection conferred by infection with viral variants, and the variant-specific risk of death if infected after vaccination. The estimates used in our simulations were chosen to be conservative with respect to these parameters, but emerging experimental data may change these results. Third, our simulation is focused narrowly on three variants, and we do not consider the generation of new, possibly more transmissible or immune-evading variants. New variants, such as Delta, which was recently detected in India, could overtake existing variants and possibly lead to more infections. Fourth, we have not included age-specific factors in our modeling, and differences in age-specific behaviors may alter the outcomes predicted here by a large margin (for example, if younger individuals have higher contact rates). Fifth, much of our attention was focused on reproductive numbers from 2–3.32, and it remains possible that, despite current reopening policies, the reproductive number for ancestral SARS-CoV-2 will remain below 2. Finally, our modeling assumes that contact rates are fixed over the entire time period, and in practice these rates are strongly impacted by current events [42, 43], as populations experiencing outbreaks of COVID-19 tend to spontaneously adopt NPIs when hospitalization and death rates climb. As a result, the findings here represent a scenario in the complete absence of rational behavior modification within the impacted population.

Nevertheless, or perhaps precisely because they represent a worst-case scenario, the simulations here provide some key lessons for the next phase of the pandemic. Crucially, our work points to the importance of keeping NPIs in place to suppress transmission of SARS-CoV-2 both during and for a period after vaccine rollout. Other recent reports have made similar observations in the context of the reopening strategies being employed in the United Kingdom [38], Portugal [40], and Italy [39]. The contact rate has a large impact on both the timing and the magnitude of subsequent waves of vaccine-resistant variants, and if the contact rate is sufficiently low, variant-driven rebounds of COVID-19 may be suppressed entirely. For example, leveraging the ability to reduce indoor transmission during the summer in the US may reduce the impact of the variants over the next few months. Thus, combining NPIs with vaccine- and naturally-induced immunity, even in the absence of herd immunity, may be a practical path forward for achieving SARS-CoV-2 suppression. Our work also suggests a metric that will yield critical information in the coming months: the absolute case counts for different variants of concern. An increase in absolute numbers of variants such as Gamma in the United States during the reopening, particularly after the bulk of the population is vaccinated, will be an early warning sign of a public health crisis.

Our results also point out two significant misconceptions in the commonly held view of the SARS-CoV-2 variants at present- the belief that the “vaccines will still work” [44–46] and the idea that the vaccines can “win the race” against the variants [47, 48] Our work shows that even relatively small reductions in immune protection (such as those reported for Gamma and WT reinfection) can have catastrophic consequences in the face of high contact and infection rates, and that high levels of pre-existing immunity will serve to select efficiently for immune-evading variants. If vaccine acceptance and efficacy against the variants under positive selection is insufficient to constrain their growth, they are likely to expand and cause significant morbidity and mortality on the population level. Thus, the vaccines may not work well enough to prevent mortality on the population level, and it is not a race. If restrictions are eased too rapidly, leading to high contact rates and high levels of viral transmission, immune-evading variants of SARS-CoV-2 are likely to expand efficiently and deterministically, leading to further waves of COVID-19. There is evidence that this scenario has already come to pass elsewhere in the world- in Manaus, after the first wave of infections in late 2020 [13, 49, 50], and in Chile and the Seychelles as of this writing [51, 52]. Our models suggest that high levels of ongoing contact in the presence of the virus will rapidly lead to disease resurgence, and this may be an intrinsic feature of the current pandemic that is unintuitive and deadly. Those who forget the recent past may be condemned to repeat it.

## Methods

### Extended S-I-R model description

To investigate SARS-CoV-2 infection dynamics and evolution after vaccine deployment, we built a deterministic extended susceptible-infected-recovered (S-I-R) epidemiological model that explicitly accounts for the spread of existing variants. The simplest extended S-I-R model we used has two competing viral genotypes circulating in the population: wild-type (WT) and variant SARS-CoV-2 (Fig 1A). This model has separate infected and recovered compartments for individuals with WT and variant infections. Importantly, vaccination and infection with WT virus completely protects individuals from infection with WT virus, but only provides partial protection from variant infection. This overestimates vaccine efficacy against WT SARS-CoV-2, as the most effective vaccines (the mRNA vaccines deployed widely in the US) have been shown to be approximately 95% effective against infection [53].

The model equations are

$$\begin{array}{cc} \frac{dS}{dt} & =\rho \left(R+R_{m}+V\right)-\left(\beta I+\beta \alpha_{m}I_{m}+z\right)S\\ \frac{dV}{dt} & =zS-(\rho +\beta \left(1-c\right)\alpha_{m}I_{m})V\\ \frac{dI}{dt} & =\beta SI-\gamma I\\ \frac{dI_{m}}{dt} & =\left(S+\left(1-c\right)\left(R+V\right)\right)\beta \alpha_{m}I-\gamma I_{m}\\ \frac{dR}{dt} & =\gamma I-(\rho +\beta \alpha_{m}\left(1-c\right)I)R\\ \frac{dR_{m}}{dt} & =\gamma I_{m}-\rho R_{m} \end{array}$$

where *S* and *V* correspond to the fractions of the population who are susceptible and vaccinated, respectively, with susceptible individuals receiving the vaccine at rate *z*. *I* and *I*_*m*_ represent the fraction of individuals who are currently infected with WT or variant SARS-CoV-2, respectively. These infected individuals eventually recover at rate *γ* and enter the recovered compartments, *R*_*w*_ and *R*_*m*_. Susceptible individuals are infected with WT virus at a rate proportional to the contact rate *β*. The variant infects susceptible individuals with an effective contact rate *βα*_*m*_, where *α*_*m*_ is the variant’s transmission advantage relative to WT. Note that there is a relationship between the contact rate *β*, the recovery period length, and the basic reproductive number *R*_0_ of the WT virus; namely, *β* = *γR*_0_. Vaccinated individuals and those who have recovered from WT infection are partially protected from variant infection and are infected at rate proportional to *βα*_*m*_(1 − *c*), where *c* < 1 is the degree of protection from variant infection conferred by WT immunity. Individuals who have recovered from the variant infection cannot be reinfected with either the WT or variant virus. Both vaccinal and natural immunity are lost at rate *ρ*.

This extended S-I-R modeling framework was further expanded to include multiple competing SARS-CoV-2 variants. In the more general case, there are *n* SARS-CoV-2 variants competing against each other and the WT virus. There are in total *n* + 1 infected and *n* + 1 recovered compartments, each corresponding to a specific viral genotype. Defining $\vec{I}$ and $\vec{R}$ as the vectors of frequencies of individuals infected and recovered from each viral genotype, we have

$$\begin{array}{cc} \frac{dS}{dt} & =\rho \sum R_{i}-zS-\left(\vec{I}\cdot B_{S}\right)S\\ \frac{dV}{dt} & =zS-\rho V-(\vec{I}\cdot B_{V})V\\ \frac{d\vec{I}}{dt} & =\left(B^{T}A\right)⊙\vec{I}-\gamma \vec{I}\\ \frac{d\vec{R}}{dt} & =\gamma \vec{I}-\rho \vec{R}-B_{R}{\vec{R}}^{T}⊙\vec{I} \end{array}$$

where ⊙ denotes element-wise multiplication. The contact rate matrix **B** describes the transmissibilities and cross infection potentials of the variants in the simulation, and has *n* + 3 rows corresponding to each of the non-infected compartments (*S*, *V*, and $\vec{R}$) and *n* + 1 columns corresponding to the infected compartments. Each entry *b*_*i*,*j*_ denotes the rate at which infected compartment *j* can infect individuals in compartment *i*. More specifically,

$$\begin{array}{c} B=\beta (\begin{array}{cccc} 1 & \alpha_{1} & \cdots & \alpha_{n}\\ 0 & \alpha_{1}c_{0,1} & \cdots & \alpha_{n}c_{0,n}\\ 0 & \alpha_{1}c_{0,1} & \cdots & \alpha_{n}c_{0,n}\\ c_{1,0} & \alpha_{1}c_{1,1} & \cdots & \alpha_{n}c_{1,n}\\ \vdots & \vdots & \ddots & \vdots \\ c_{n,0} & \alpha_{1}c_{n,1} & \cdots & \alpha_{n}c_{n,n} \end{array}) \end{array}$$

where *α*_*i*_ is the transmission advantage of variant *i* and *c*_*i*,*j*_ is the cross immunity to infection with variant *j* conferred by previous infection with variant *i*, where variant 0 is WT. The values for *α*_*i*_ are the transmissibilities relative to WT given in S1 Table. The values for *c*_*i*,*j*_ are 0 for *i* = *j* or when variant *j* does not have an arrow pointing to variant *i* in the schematic in Fig 2A; otherwise 1 − *c*_*i*,*j*_ is the cross immunity value for variant *j* in S1 Table. The first row (also defined as **B**_*S*_) corresponds to the rates at which the WT and variants infect susceptible individuals. Similarly, the second row (also defined as **B**_*V*_) corresponds to the rates at which the WT and variants infect vaccinated individuals. The remaining rows of this matrix are defined as **B**_*R*_, and corresponds to the rates at which the WT and variants infect recovered individuals. The cross immunity relationships shown in Fig 2A were used to populate the entries of **B**. The column vector **A** has entries *a*_1_ = *S*, *a*_2_ = *V*, and *a*_*i*+2_ = *R*_*i*_ for *i* = 0, …, *n*.

To simulate both versions of the model, the above systems of differential equations were numerically solved using scipy.integrate (version 1.4.1) in Python. Parameter values, initial conditions, and references used for the simulations are provided in S1 and S2 Tables.

### Estimation of total fatalities due to COVID-19

The total number of deaths due to COVID-19 during the first year after the starting point of the simulations was estimated by using the simulation results to calculate the number of COVID-19 cases occurring in previously unexposed or previously-infected/vaccinated individuals separately. Individuals who were not previously exposed to SARS-CoV-2 were assumed to have an infection fatality ratio (IFR) of 0.68% [33].

We estimated the IFR for vaccinated/immune individuals relative to unexposed individuals using the Pfizer-BioNTech mRNA vaccine efficacy data published in [34]. The IFR is defined as the number of fatalities due to infection divided by number of infected individuals. However, it is difficult to directly estimate the IFR for vaccinated individuals from raw incidence data from large-scale observational COVID-19 vaccine studies such as [34] for two reasons. First, these studies only record confirmed cases, so we are limited to estimating the case fatality ratio, which often is not an accurate proxy for the IFR [54]. Second, the IFR for COVID-19 depends strongly on age. Since vaccinated individuals have a different age distribution than unvaccinated individuals in [34], we must correct for these differences in age when assessing the relative IFR of vaccinated individuals. To solve both of these problems, we used the age- and sex-adjusted vaccine efficacy data in [34] to calculate the IFR of vaccinated individuals relative to unvaccinated individuals, and multiplied this relative IFR with a previous estimate of the overall unvaccinated IFR (0.68%) taken from a meta-analysis of IFRs estimated from a number of intensive dedicated surveillance efforts [33]. This relative IFR (IFR_rel_) is defined as a ratio of the true vaccinated (IFR_*v*_) and unvaccinated (IFR_*u*_) IFRs as follows:

$$\begin{array}{cc} {\text{IFR}}_{\text{rel}} & =\frac{{\text{IFR}}_{v}}{{\text{IFR}}_{u}}\\ & =\frac{\text{number}\;\text{of}\;\text{vaccinated}\;\text{fatalities}/\text{number}\;\text{of}\;\text{vaccinated}\;\text{infections}}{\text{number}\;\text{of}\;\text{unvaccinated}\;\text{fatalities}/\text{number}\;\text{of}\;\text{unvaccinated}\;\text{infections}}\\ & =\left(\frac{\text{rate}\;\text{of}\;\text{vaccinated}\;\text{fatalities}}{\text{rate}\;\text{of}\;\text{unvaccinated}\;\text{fatalities}}\right)\left(\frac{\text{rate}\;\text{of}\;\text{unvaccinated}\;\text{infections}}{\text{rate}\;\text{of}\;\text{vaccinated}\;\text{infections}}\right)\\ & =\frac{\text{vaccine}\;\text{fatality}\;\text{rate}\;\text{ratio}}{\text{vaccine}\;\text{infection}\;\text{rate}\;\text{ratio}}. \end{array}$$

The rate ratios are defined as the incidence rate of the event (death or infection) for vaccinated individuals divided by the incidence rate in unvaccinated individuals. In [34], the adjusted vaccine efficacy against all SARS-CoV-2 infections is reported as (1 − vaccine infection rate ratio) and the adjusted vaccine efficacy against COVID-19-related death (corrected for age and sex) is reported as (1 − vaccine fatality rate ratio). Therefore, we estimated IFR_rel_ as $\frac{1-\text{efficacy}\;\text{against}\;\text{death}}{1-\text{efficacy}\;\text{against}\;\text{infection}}=0.54$. The overall IFR for vaccinated individuals is 0.54 * 0.68% = 0.36%.

## Supporting information

S1 Table Parameter values for transmissibility and cross immunity for SARS-CoV-2 variants. (PDF)

S2 Table Other parameter values used to simulate SARS-CoV-2 spread. (PDF)

S1 Dataset Minimal simulation results dataset used in this study. (XLSX)
