# Supplementary material for: Rapid relaxation of pandemic restrictions after vaccine rollout favors growth of SARS-CoV-2 variants: A model-based analysis
Source: PLoS One. 2021 Nov 24;16(11):e0258997. doi: 10.1371/journal.pone.0258997 (PMC8612578; doi:10.1371/journal.pone.0258997)
Supplement: S1 Table — (PDF) [file pone.0258997.s001.pdf]

S1 Table. Parameter values for transmissibility and cross immunity for SARS-CoV-2 variants.

Variant	Transmissibility relative to WT	Cross immunity with WT	% of US infections as of 4/10/21
Alpha	1.59 [1]	89.5% [2]	59.6%
Beta	1.5 [3]	75.0% [2]	1.0%
Gamma	2.0 [4]	68.0% [4]	3.7%

Transmissibility and cross immunity estimates were taken directly from the references cited in the table. All variant frequency data was taken from the CDC COVID Data Tracker [5].

References for S1 Table

[1] Davies NG, Abbott S, Barnard RC, Jarvis CI, Kucharski AJ, Munday JD, et al. Estimated transmissibility and impact of SARS-CoV-2 lineage B.1.1.7 in England. Science. 2021;doi:10.1126/science.abg3055.

[2] Abu-Raddad LJ, Chemaitelly H, Butt AA. Effectiveness of the BNT162b2 Covid-19 Vaccine against the B.1.1.7 and B.1.351 Variants. New England Journal of Medicine. 2021;0(0):null. doi:10.1056/NEJMc2104974.

[3] Pearson CAB, Russell TW, Davies N, Kucharski AJ, working group CC, Edmunds WJ, et al. Estimates of severity and transmissibility of novel SARS-CoV-2 variant 501Y.V2 in South Africa; 2021. Available from: `https://cmmid.github.io/topics/covid19/sa-novel-variant.html`.

[4] Faria NR, Mellan TA, Whittaker C, Claro IM, Candido DdS, Mishra S, et al. Genomics and epidemiology of the P.1 SARS-CoV-2 lineage in Manaus, Brazil. Science. 2021;doi:10.1126/science.abh2644.

[5] CDC. COVID Data Tracker;. Available from: `https://covid.cdc.gov/covid-data-tracker`.
