# Supplementary material for: Rapid relaxation of pandemic restrictions after vaccine rollout favors growth of SARS-CoV-2 variants: A model-based analysis
Source: PLoS One. 2021 Nov 24;16(11):e0258997. doi: 10.1371/journal.pone.0258997 (PMC8612578; doi:10.1371/journal.pone.0258997)
Supplement: S2 Table — (PDF) [file pone.0258997.s002.pdf]

S2 Table. Other parameter values used to simulate SARS-CoV-2 spread.

Parameter	Value	Reference
Initial fraction of population vaccinated	50%	[1]
Daily vaccine uptake rate (% susceptibles vaccinated/day)	1%	[1] (upper bound)
Initial fraction of population previously infected	30%	[2]
Initial fraction of population currently infected	0.13%	$\frac{\text{(current case count)(mean infection duration)}}{\text{total US population}}$
Mean infection duration	10 days	[3]
Mean duration of immunity	18 months	[4] (upper bound)
R_0 of WT SARS-CoV-2	3.32	[5]
Total population size	300 million	approximate size of US population

References for S2 Table

[1] U.S. COVID-19 vaccine tracker: See your state's progress;. Available from: `https://www.mayoclinic.org/coronavirus-covid-19/vaccine-tracker`.

[2] CDC. Cases, Data, and Surveillance; 2020. Available from: `https://www.cdc.gov/coronavirus/2019-ncov/cases-updates/burden.html`.

[3] Rocklöv J, Sjödin H, Wilder-Smith A. COVID-19 outbreak on the Diamond Princess cruise ship: estimating the epidemic potential and effectiveness of public health countermeasures. J Travel Med. 2020;27(3). doi:10.1093/jtm/taaa030.

[4] Isho B, Abe KT, Zuo M, Jamal AJ, Rathod B, Wang JH, et al. Persistence of serum and saliva antibody responses to SARS-CoV-2 spike antigens in COVID-19 patients. Science Immunology. 2020;5(52). doi:10.1126/sciimmunol.abe5511.

[5] Alimohamadi Y, Taghdir M, Sepandi M. Estimate of the Basic Reproduction Number for COVID-19: A Systematic Review and Meta-analysis. J Prev Med Public Health. 2020;53(3):151–157. doi:10.3961/jpmph.20.076.
